# Prognostic and Diagnostic Value of Endocan in Kidney Diseases

**DOI:** 10.1155/2022/3861092

**Published:** 2022-03-14

**Authors:** Elisabeth Samouilidou, Virginia Athanasiadou, Eirini Grapsa

**Affiliations:** ^1^Department of Biochemistry, “Alexandra” Hospital, Athens, Greece; ^2^Renal Unit, “Aretaeio” Hospital, Athens, Greece

## Abstract

Endocan, previously called endothelial cell-specific molecule-1, is a soluble proteoglycan that is predominantly expressed in vascular endothelial cells of the lungs and kidneys. It is upregulated by proinflammatory cytokines and plays a critical role in inflammatory, proliferative, and neovascularization processes. The utility of endocan as a biomarker in a wide spectrum of diseases is being increasingly acknowledged. In this review, we summarize the current evidence concerning the role of endocan in kidney diseases, with emphasis on its prognostic and diagnostic value. It seems that the determination of plasma endocan levels may provide useful prognostic information in many types of renal failure such as chronic kidney disease, IgA nephropathy, and diabetic nephropathy. Endocan could additionally improve the early diagnostic evaluation of acute kidney disease, chronic renal allograft injury, and acute rejection after kidney transplantation, thus contributing to endothelial cell injury monitoring in a timely manner.

## 1. Introduction

Endocan is a soluble proteoglycan that was first discovered in human umbilical vein cells (HUVECs) [1]. It was initially called endothelial cell-specific molecule 1 (ESM-1) and later renamed endocan because it is mainly produced in endothelial cells [2, 3]. Endocan is predominantly expressed in the lungs and kidneys and in the gastrointestinal tract, thyroid gland, lymph node, liver, brain and is secreted in the bloodstream. Its secretion is upregulated by proinflammatory cytokines, such as interleukin 1*β* (IL-1*β*) and tumor necrosis factor alpha (ΤNF-*α*) and promotes the activation of inflammatory signaling pathways [1, 3]. Additionally, endocan interferes with the interaction between the lymphocyte-function associated antigen-1 (LFA-1) and the intracellular cell adhesion molecule-1 (ICAM-1), mediating leukocyte adhesion and migration from the blood into the injured tissues [2].

In the last two decades, there has been convincing documentation about the implication of endocan in several inflammatory and endothelial disorders, linked to pulmonary dysfunctions [4–6], cardiovascular events [7–9], kidney diseases [10–12], sepsis [13, 14], and cancer [15–17]. This paper aims to summarize recent evidence concerning the role of endocan in the development and the progression of kidney diseases, focusing on its prognostic and diagnostic potential.

## 2. Biological Characteristics and Functions of Endocan

### 2.1. Structure, Expression, and Regulation

Endocan in humans is encoded by the ESM-1 gene, located in the long arm of chromosome 5 at position q11.2. The ESM-1 gene covers 12 kilobases and is organized into three exons. Ιn the mature form, human endocan is a 50 kDa soluble dermatan sulphate (DS) proteoglycan, which consists of a protein core of 165 amino acids and a glycosaminoglycan (GAG) type polysaccharide chain, covalently attached to the protein on serine 137 [1, 18]. The protein core contains an N-terminal cysteine-rich region (110 amino acids) with an endothelial growth factor-like region, a phenyl alanine-rich domain, and a C-terminal region. The high percentage of sulfated components in the protein core contributes to the binding properties of endocan. The GAG chain includes 32 disaccharide residues, consisting of an amino sugar (most often N-acetyl glucosamine or N -acetyl galactosamine) and uronic acid, mainly responsible for conformational flexibility. It seems that the multifunctionality of endocan arises from its structure and involves the interaction of the GAG chain and the protein core with various ligands [19].

In contrast to most chondroitin/DS-containing proteoglycans, which are either extracellular matrix or cell membrane-associated, endocan is a secreted proteoglycan. It is expressed by the vascular endothelial cells, mainly in the lungs and to a lesser extent in the kidneys. Besides, it is detectable in the gastrointestinal tract, liver, brain, thyroid gland, lymph node, and skin but absent in the breast and large vessels or spleen [1, 20]. Endocan is found in the bloodstream and urine in healthy individuals and in pathological states, such as cardiovascular disease, chronic kidney disease, sepsis, acute respiratory distress syndrome, and several types of cancer [8, 12, 16, 21–23].

The expression and secretion of endocan are both significantly enhanced in vitro by the proinflammatory cytokines TNF-*α* and IL-1*β* [1, 3]. In contrast, the spontaneous, as well as TNF*α*-induced, secretion of endocan is strongly inhibited by IFN-*γ* (interferon -*γ*). The regulation of endocan by the above cytokines was additionally observed at the protein level. Proangiogenic factors such as VEGF-A (vascular endothelial growth factor- A) and FGF-2 (fibroblast growth factor -2) also upregulate endocan mRNA and protein expression in endothelial cells, likewise in high-grade glioma cells [24] and in human renal cancer cells, whereas PI3K (phosphatidylinositol 3 kinase) has inhibitory effects [25].

In the human kidney, endocan is expressed not only by the glomerular endothelium but by epithelial cells from distal and proximal tubules too and is regulated by proinflammatory cytokines. It is catabolized by proteolytic cathepsin G derived from activated neutrophils to a major fragment of 14 KDa, named p14, which is a cysteine-rich part that could be eliminated through glomerular filtration [21, 23].

### 2.2. Functions

Recent data corroborate the significance of endocan in inflammatory, proliferative, and neovascularization processes. In HUVECs under intermittent hypoxia status, endocan promotes the expression of cell adhesion molecules ICAM-1 (intercellular adhesion molecule-1) and VCAM-1 (vascular cell adhesion molecule-1) through hypoxia-inducible factor-1 alpha (HIF-1*α*)/VEGF pathway. This plays a critical role in enhancing adhesion between monocytes and endothelial cells and might contribute to endothelial dysfunction [26]. Furthermore, endocan elicits severe vascular inflammatory responses in cellular and animal experimental models of sepsis [27]. In HUVECs, it increases the release of chemokines interleukin-8 and monocyte chemotactic protein-I [MCP-I]), induces the expression of ICAM-1, VCAM-1, and E-selectin, and stimulates mitogen-activated protein kinase protein (MAPK) signaling pathway and NF-*κ*B (nuclear factor kappa B). Additionally, endocan in high serum concentrations enhances leukocyte migration and induces endothelial cytoskeletal rearrangement, leading to cellular contraction and alteration of cellular permeability.

On the other hand, endocan seems to have anti-inflammatory activity as well. In hypertensive patients characterized by high serum ICAM-1 concentration, endocan released by activated endothelium could compete with ICAM-1 for the integrin LFA-1, which is the alpha L/beta2 leukocyte ligand for ICAM-1. Thus, endocan could exert a protective effect by inhibiting leukocyte adhesion and disturbing cell transmigration to sites of inflammation [28].

Endocan, moreover, increases in a dose-dependent way, the hepatocyte growth factor/scatter factor (HGF/SF)-mediated proliferation of human embryonic kidney cells [2, 16] and promotes the mitogenic and promigratory activity of both VEGF-A and -C on cultured lymphatic endothelial cells, constituting a novel mediator of lymphangiogenesis and a potential therapeutic target for cancer therapy [29]. Its marked cytoplasmic expression inside tumor vessels in different types of highly vascularized tumors such as cancer of the lung [15, 30], brain [24, 31], kidney [25, 32], liver [33, 34], pituitary [35], and bladder [36] was suggested to correlate with tumor aggressiveness and progression [16]. The presence of endocan in hyperplastic endothelial cells in high-grade gliomas, principally activated by VEGF-A and TGF*β*2 signaling pathway [24, 37] and in tumor cells and vessels located in the tumor stroma of human renal carcinoma [25], might be indicative of the role of endocan in the growth of renal tumor cells and in tumor vascularization. The specific expression of endocan in endothelial tip cells, involved in vascular growth and neoangiogenesis, may be, however, suggestive of its utility for monitoring treatment with antiangiogenic factors [38].

## 3. Endocan and Kidney Diseases

Endothelial cell damage or injury is constantly associated with many clinical conditions in patients with kidney diseases, such as thrombosis, hypertension, renal failure, atherosclerosis, and sepsis [9–41]. In chronic kidney disease, traditional risk factors cannot explain the high prevalence of the cardiovascular disease. Therefore, factors related to endothelial dysfunction have increasingly been studied [39]. Endocan, playing a significant role in the regulation of cell adhesion and inflammatory disorders, has been shown to provide promising utilities in various kidney disease entities, such as acute kidney injury [42, 43], chronic kidney disease [44, 45], hemodialysis [46], peritoneal dialysis [47, 48], renal transplantation [49, 50], immunoglobulin A nephropathy [51], autosomal dominant polycystic kidney disease [52], and diabetic nephropathy [53, 54]. It constitutes a potential biomarker in the prognosis and diagnosis of kidney diseases; however it has, so far, significant limitations (Table 1).

**Figure 1:**
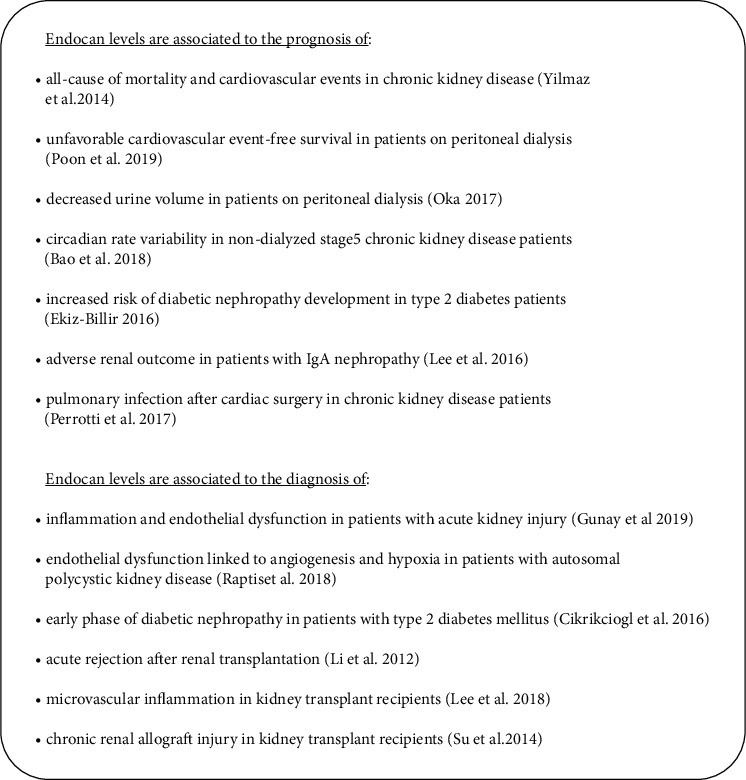
The prognostic and diagnostic value of endocan in kidney diseases.

In this study, the main findings showing the role of endocan in kidney diseases are described below.  Figure 1 delineates the prognostic and diagnostic value of endocan as emerged from the conclusions of recent literature. The most important results concerning the role of endocan in the development and the progression of kidney diseases are summarized in Table 2.

### 3.1. Endocan and Acute Kidney Injure (AKI)

Acute kidney injury (AKI) is defined as any of the following: increase in serum creatinine by ≥0.3 mg/dL within 48 hours or increase in serum creatinine to ≥1.5 times baseline within the last 7 days or urine output less than 0.5 mg/kg/h for 6 hours [61]. The diagnostic methods for AKI are mainly based on serum creatinine measurement; however, the decreased sensitivity and specificity of this marker, which does not always reflect the extent of destruction of renal parenchyma, has led to the evaluation of alternative markers associated with inflammation and endothelial dysfunction, such as endocan [42, 43].

Endocan plasma levels were firstly determined in relation to acute renal failure in a group of 96 patients with acute respiratory distress syndrome (ARDS) who were not receiving renal replacement therapy (RRT) at diagnosis during the ICU stay [55]. In this post hoc study of prospectively collected data, it was found that patients with ARDS needing RRT had significantly higher endocan and creatinine levels, but also higher values of APACHE II (Acute Physiologic Assessment and Chronic Health Evaluation II) and SOFA (Sequential Organ Failure Assessment) scores compared to patients who did not need RRT. In addition, endocan, when combined with blood creatinine levels, had better prognostic value in the prediction of the need for RRT in comparison to creatinine alone. Gunay et al. conducted a study with patients with AKI, not having other acute or chronic diseases or using any kind of medications (*n* = 39) to evaluate serum endocan levels in comparison to healthy individuals [42]. They found that endocan levels were significantly higher in the study group than in the controls. The receiver operating characteristic (ROC) analysis showed high sensitivity and specificity of endocan (59% and 76.3%, respectively) in the diagnosis of AKI. The authors remarked that the mechanism responsible for the increased serum endocan level in patients with reduced kidney function is unclear. Therefore, it could be hypothesized that increased serum levels could be a consequence of elevated production or a decreased renal clearance, feasibly through the disrupted glomerular basement membrane. However, the authors concluded that an increased endocan level in patients with AKI is a significant marker of inflammation and endothelial injury. Gaudet et al. performed a post hoc analysis based on data from previous research of severe septic patients (*n* = 99) to study the impact of acute renal failure on plasmatic levels of p14, a major catabolite of endocan [56]. The authors determined the plasma endocan cleavage ratio (ECR) which was calculated as plasma p14/(endocan + p14) ratio and measured ECRs at baseline and 24,48 and 72 h after admission to the ICU. The results showed that renal SOFA was the only component of the SOFA scale associated with higher ECR at baseline. Over 72 h, patients with a baseline renal SOFA at 4 had significantly higher ECR than those with baseline SOFA<4. These findings suggest that circulating concentrations of p14 could relate to the severity of the acute renal failure. However, it is preferable to measure p14 in urine rather than in blood, as p14 could be eliminated through glomerular filtration due to its small molecular weight and the absence of a polyanionic glycanic chain on its protein core. Interestingly, in a theoretical study, an additional role was ascribed to serum endocan in AKI by means of an algorithmic guideline approach [43]. It was suggested that serum endocan, possibly signifying endothelial damage, could be used to discriminate glomerular/vasculoendothelial injury from tubular injury and, even more, differentiate AKI from the acute presentation of CKD. Nevertheless, experimental studies are needed to further support the above hypotheses.

### 3.2. Endocan, Chronic Kidney Disease (CKD), and Dialysis

Chronic kidney disease (CKD) is defined as abnormalities of kidney structure or function, present for more than three months, with implications for health. CKD is classified based on cause, glomerular filtration rate (GFR) category (G1-G5), and albuminuria category (A1-A3) [62]. CKD is accompanied by an irreversible and progressive loss of kidney function, which is associated with a decreased GFR of less than 90 mL/min/1.73 m^2^ or damaged kidney structure. CKD patients have accelerated atherosclerosis, elevated risk of thrombotic disorders, and increased mortality. Furthermore, there is evidence of endothelial activation and injury that may end in a dysfunctional state [63]. Treatment in CKD can be conservative (for patients without indication for dialysis, usually those with GFR above 15 ml/minute) or replacement therapy (hemodialysis, peritoneal dialysis, and kidney transplantation). The aim of treatment is to slow down the progression of kidney failure and along with the complications, such as anemia, bone diseases, and cardiovascular disease Endocan could play a crucial role in the development and progression of CKD since it takes part in endothelial cell activation and vascular inflammation processes, and may constitute a novel noninvasive marker [64].

Yilmaz et al. studied plasma endocan levels in nondialyzed CKD patients at stage1−5 (*n* = 251) in relation to inflammation, endothelial dysfunction, cardiovascular incidence, and overall survival [11]. They found that plasma endocan concentration correlated with estimated GFR (eGFR), different markers of inflammation (pentaxin 3 and high-sensitivity C-reactive protein (hs-CRP)), and vascular abnormalities (flow-mediated vasodilation (FMV) and carotid intima thickness (CIMT)). Patients with CKD had increased endocan levels compared with controls, with values progressively higher across stages of CKD. Plasma endocan concentrations correlated negatively with FMV and positively with hs-CRP and CIMT on multivariate analysis. On univariate Cox survival analysis, increasing endocan levels were associated with an elevated risk of death and cardiovascular events (CVE). In multivariate Cox models, after adjustment for traditional and renal-specific risk factors, endocan levels were independently associated with all-cause mortality and CVE. Besides the strong prognostic value of endocan for mortality and CVE, it was observed that adding endocan to a prediction model based on common and nontraditional risk factors improved the model prediction capability for fatal and nonfatal CVE. Pawlak et al. conducted a cross-sectional study in nondialyzed CKD patients (*n* = 53) with and without cardiovascular disease (CVD), aiming to evaluate plasma endocan, soluble ICAM-1 (sICAM-1), soluble VCAM-1 (sVCAM-1), markers of inflammation (hs-CRP, interleukin-6, TNF-*α*), and their interrelations [10]. They found that plasma endocan, sICAM-1, sVCAM-1, and hs-CRP levels were significantly higher in CKD patients than in controls and also significantly higher in CKD patients with CVD than in those without CKD. However, unlike the previous [11] and most of the studies, no correlation was found herein between endocan and kidney function markers (eGFR, creatinine, and urea concentrations), suggesting that endocan increased levels are rather the result of intensified secretion than reduced renal clearance. This suggestion was equally supported by the fact that endocan was significantly higher in patients with CVD than those without CVD, whereas the markers of the kidney function were similar in both of these groups. Moreover, a positive correlation between endocan and sICAM-1 and sVCAM-1 concentrations was exclusively shown in a subgroup with CVD. In CKD patients with CVD, a low % percentage of lymphocytes followed by increased endocan was identified as the independent variables significantly associated with increased sICAM-1 and sVCAM-1 concentrations. Consistent with those findings, the authors hypothesize that the possible mechanism explaining the increase in serum endocan in uremic patients with CVD rests on the high concentrations of sICAM-1/sVCAM-1 under the condition of exacerbated inflammation that could cause high adherence of lymphocytes into endothelium, thereby reducing circulating lymphocytes. This induces the release of endocan by activated endothelial cells that, in turn, could constitute a compensatory, protective mechanism disrupting the lymphocyte adhesion into endothelium. However, a limitation mentioned was that functional tests with respect to the endothelial function in those patients were also needed to confirm the above hypotheses [10]. Perrotti et al. followed a pilot study with patients with preoperative CKD (*n* = 166) who underwent cardiac surgery in order to evaluate the relevance of plasma endocan levels for predicting pulmonary infection after cardiac surgery [58]. Blood samples were tested at 4 time points (preoperatively and 6,12, and 24 h after the end of the surgery) and plasma endocan, procalcitonin, and CRP levels were compared. At 6 h, the patients with pulmonary infection had significantly higher levels than patients without pulmonary infection. A ROC analysis showed 80% sensitivity and 100% specificity for endocan to predict pulmonary infection, with a cutoff value of 15.9 ng/mL. Interestingly, the time saved by assessment of the endocan concentration compared to a clinical diagnosis of pulmonary infection was 47 h. Bao et al. investigated the link between serum endocan and circadian heart rate (HR) variability [57], which has been shown to be an important risk factor for cardiovascular morbidity and mortality in CKD patients [65]. In this cross-sectional study enrolled with nondialysis stage 5 CKD patients (*n* = 54) [57], it was found that patients with low endocan levels had significantly lower resting HR than those with high endocan levels. Night/day HR ratio was positively correlated with serum endocan in univariate analysis. In multivariate regression analysis of predictors for night/day HR ratio, HR ratio was independently predicted by serum endocan level and hypertension history. The above findings suggest that increased serum endocan level may be useful for predicting circadian heart rate variability in nondialysis stage 5 CKD patients.

The association between serum endocan and dyslipidemia, a common disorder observed in CKD patients on hemodialysis, was examined in our previous study [46]. CKD patients (*n* = 105) were divided into nondialyzed and hemodialysis (HD) subgroups. Endocan was analyzed in relation to lipid profile and also to two HDL-linked members of the paraoxonase (PON) family, PON1 and PON3, which contribute to HDL-related antiatherogenic properties. It was found that endocan levels were significantly higher in HD patients than in nondialyzed patients and controls. Endocan levels correlated positively with total cholesterol and LDL-C in both studied groups and inversely with HDL-C and PON1 concentrations in HD patients. Multiple regression analysis between endocan and the above lipid parameters in the total of patients revealed that endocan was independently associated only with PON1. This probably indicates that the increase in serum endocan levels in CKD patients may be associated with the decrease in PON1 concentration, irrespective of lipid alterations produced by atherosclerosis development. McMillan et al. studied the role of endothelial, renal, and inflammatory biomarkers (such as endothelin, endocan, kidney injury molecule-1 (KIM-1), N-terminal probrain natriuretic peptide (NT-proBNP), glycated hemoglobin, interleukin-18, and neutrophil gelatinase-associated lipocalin) in the pathogenesis of heart failure (HF) in 90 patients with stage 5 chronic kidney disease undergoing maintenance hemodialysis (CKD5-HD) [66]. The results demonstrated statistically significant differences between CKD5-HD patients and healthy controls for the majority of those biomarkers, including endocan. However, in contrast to Yilmaz et al. [11], who showed association with CVE, herein the comparison between HF (+) and HF (−) patients did not show any difference regarding endocan. The authors concluded that plasma NT-proBNP and KIM-1 seem solely to contribute to the pathogenesis of heart failure in patients on hemodialysis.

Regarding peritoneal dialysis (PD), Oka et al. proved that endocan has prognostic power for the decrease in urine volume [48], which is an often problem in PD patients, associated with the decline in residual renal function, resulting in fluid overload, hypertension and increased risk for cardiovascular events [67]. In this cohort study with 21 enrolled subjects, serum endocan levels in PD patients were higher than controls and higher in the rapid-decrease subgroup (decrease of urine volume>200 mL/year) than in the slow-decrease subgroup (decrease of urine volume<200 mL/year). Serum endocan was positively correlated with proteinuria level, serum creatinine, and TNF-*α*, but not with urine volume at baseline. Nevertheless, multiple linear regression analysis showed that the serum endocan level and proteinuria level at baseline were independent predictors for the extent of the decline in urine volume. Poon et al. examined the relationship between serum endocan level and clinical outcome of PD patients (*n* = 193) [47]. Serum endocan levels, stratified into tertile 1 (lowest) to 3 (highest), markers of nutritional status, and markers affecting vascular physiology and peritoneal transport were measured. The results revealed that patients with higher serum endocan levels in tertile 3 had lower serum albumin levels, higher carotid-femoral pulse wave velocity (PWV), and higher CRP than tertiles 2 and 1. Furthermore, there was a progressive decrease in the subjective global assessment scale and an increase in malnutrition -inflammation score from tertile 1 to 3. Multivariate Cox regression analysis showed that serum endocan level was an independent predictor of cardiovascular event-free survival. The authors deduced that high serum endocan level is associated with unfavorable nutritional, arterial, and inflammatory conditions in PD patients and that in patients with suboptimal blood pressure control, higher serum endocan is related to worse cardiovascular outcomes.

### 3.3. Endocan and Kidney Transplantation

Kidney transplantation (KT) is one of the most effective options for the treatment of chronic kidney disease. Patients who receive renal transplants have better survival rates than those who undergo dialysis; however, progressive deterioration of graft function constitutes a major clinical problem. The persistent posttransplantation alloimmune reactivity between recipient leukocytes and endothelial cells in renal allografts may lead to injury, inflammation, along with endothelial dysfunction associated with graft loss [68]. Different studies focus on the determination of markers that would facilitate the early diagnosis of graft deterioration and acute rejection. In 2012, Li et al. evaluated the impact of dynamic monitoring of endocan expression in the serum and renal allograft tissues of sixty kidney transplant recipients followed up preoperatively and after acute rejection [59]. They found that ESM-1 mRNA and protein expression were significantly increased in patients with acute rejection compared to patients with normal allograft function and allograft dysfunction from other causes. The antirejection treatment decreased ESM-1 mRNA expression. In peripheral blood, ESM-1 mRNA was elevated in patients with acute vascular rejection compared to those with acute cellular rejection. The sources of ESM-1 were mainly from the damaged endothelial tissue of the donor's kidney, including vascular endothelium and glomerular endothelial cell. The above data demonstrated that endocan mRNA and protein expression may reflect the status of endothelial cell injury of renal allografts. The authors suggest that endocan may serve as a sensitive and specific marker for acute rejection after renal transplantation. The prognostic value of endocan for acute rejection could even increase when combined with the determination of urine HLA-DR + lymphocytes. Su et al. conducted a cross-sectional study with renal transplants (RT) recipients (*n* = 97) to determine whether serum endocan was related to chronic renal allograft injury [50]. They observed that serum endocan levels correlated with CKD stage and higher endocan levels in more advanced CKD stages. Patients with higher serum endocan had higher creatinine and lower eGFR levels than those with lower serum endocan in the 3-month follow-up. Linear regression analysis showed a significant correlation between endocan and TNF-*α*. The role of the proinflammatory cytokine TNF-*α* in posttransplantation alloimmune reactivity and endocan expression was further studied in HUVECs culture. There was a substantial increase in endocan and transforming growth factor (TGF)-*β*1 production in the presence of TNF-*α*, while IL-10 progressively decreased over time. This phenomenon was attributed to the bidirectional role of TNF-*α*, which activates the recruitment of inflammatory cells at the damaged endothelium through the endocan effect, while at the same time exerting anti-inflammatory activity through TGF-*β*1 and IL-10. The authors concluded that endocan may have potential as a long-term indicator of chronic renal allograft injury in RT recipients. De Souza et al. evaluated serum endocan concentrations in 62 pediatric patients 6–24 months after KT and assessed their relationship with hypertension and loss of renal function [60]. Hypertension constitutes major cardiovascular comorbidity that can follow renal transplantation in pediatric patients [69]. De Souza et al. found that the endocan levels were significantly elevated in the pediatric patients who had hypertension and loss of renal function compared with that in pediatric RT patients who did not have either of these conditions. The endocan levels were inversely correlated with eGFR and positively with systolic blood pressure and pulse pressure. The ROC curve analysis demonstrated that an endocan cutoff concentration of 7.0 ng/mL could identify pediatric RT patients with both hypertension and loss of renal function with a sensitivity of 100% and a specificity of 75%. Lee et al. evaluated the clinical relevance of endocan as a marker of microvascular inflammation in patients who underwent KT, having different etiologies of allograft dysfunction and various pathologic scores (*n* = 203) [12]. They found that recipients experiencing acute antibody-mediated rejection (ABMR) have significantly higher levels of plasma and urinary endocan concentrations than patients with normal pathology, acute tubular necrosis, acute pyelonephritis, BK virus associated nephropathy, and T-cell mediated rejection. Scores of glomerulitis and peritubular capillaritis, which characterize microvascular inflammation, were significantly elevated in patients with higher plasma and/or urinary endocan levels, whereas neither tubulitis nor total interstitial inflammation scores were associated with plasma or urinary endocan quartiles. Moreover, patients exhibiting acute ABMR and high plasma and urinary endocan levels showed significantly worse renal survival. The authors inferred that plasma and urinary endocan levels not only may serve as diagnostic tools for vascular inflammation but also could effectively discriminate ABMR from other allograft pathologies. However, the authors mentioned as a limitation of this study the impact of certain medications (angiotensin receptor blockers, calcium channel blockers, and statins) on the endothelial integrity and the need to investigate the effect of immunosuppressive agents on plasma and urinary endocan levels. In the study of Malyszko et al., serum endocan level was evaluated in sixty-three KT recipients with stable graft function in correlation with other markers of endothelial damage (ICAM and VCAM), markers of inflammation (CRP and IL-6), and markers of kidney function (creatinine, eGFR and cystatin C) [49]. The results demonstrated that endocan level was significantly higher in KT patients than in healthy controls and was related to renal function (negative correlation with eGFR and positive correlation with creatinine), time after transplantation (negative correlation), as well as endothelial dysfunction (positive correlation with ICAM and VCAM). In multivariate analysis, the predictors of endocan levels were creatinine, ICAM, and VCAM, predicting 59% of the variability. The authors suggested that serum endocan level in KT recipients could indicate the beginning of pathological processes stemming from endothelial dysfunction and injury.

### 3.4. Endocan and Diabetic Nephropathy

Diabetes mellitus (DM) is a group of heterogeneous metabolic disorders characterized by hyperglycemia, which can cause many serious macrovascular complications, such as cardiovascular disease, stroke, and peripheral artery disease, as well as microvascular problems, such as neuropathy, retinopathy, and also chronic kidney disease. Diabetic nephropathy is a major cause of end-stage renal disease that may be related to an imbalance between the expression of proangiogenic growth factors and antiangiogenic molecules, leading to angiogenesis and endothelial damage [53]. In a preliminary study, Arman et al. examined the effect of glycemic regulation on endocan levels in diabetic patients with type 2 DM, who did not have other inflammatory diseases (*n* = 77) [70]. After three months of lifestyle modifications and pharmacological treatment of diabetes, the authors found that the levels of endocan, hemoglobulin A1c, and urinary albumin-to-creatinine ratio (UACR) were significantly reduced compared with the baseline levels. A multivariate regression analysis revealed that only the decrease of UACR was independently correlated with the decrease of endocan in diabetic patients following antihyperglycemic treatment. It was concluded that the decrease in endocan concentrations might be associated with improved glycemic control and reductions in UACR. Ekiz-Bilir et al. performed a study with type 2 DM patients (*n* = 96) with and without diabetic nephropathy to assess the predictive role of endocan and endoglin as markers of angiogenesis and diabetic nephropathy progression [53]. Endoglin is another glycoprotein expressed in endothelial cells required for vascular development and angiogenesis [71]. It was found that serum endocan and endoglin levels of diabetic patients were higher than those of the controls [53]. Endocan levels were increased in nephrotic diabetic patients compared to normoalbuminuric diabetic patients and controls. On the contrary, there was no statistically significant difference in endoglin levels between the above groups. In view of the importance of early recognition of microvascular complications in patients with diabetes mellitus at high risk for diabetic nephropathy, the authors emphasized that endocan could be a reliable marker of nephropathy development in those patients. Cikrikcioglu et al. studied the association between serum endocan levels and UACR in patients with type 2 DM (*n* = 137) divided into normoalbuminuria, microalbuminuria, and macroalbuminuria groups [54]. Surprisingly and in contrast to the above studies, they found that although three groups were similar in terms of serum creatinine level and eGFR, serum endocan level was lower in patients with macroalbuminuria than in normoalbuminuric and microalbuminuric patients. Besides, there was a bivariate negative correlation between UACR and serum endocan levels and a positive correlation between serum endocan levels and systolic blood pressure. The authors hypothesized that the unexpected obtained results could be explained on the basis of the VEGF-A related mechanism. According to this hypothesis, in the early phases of diabetic nephropathy, hyperglycemia leads to abnormal angiogenesis and enhancement of kidney vascular permeability through stimulation of VEGF-A [72], a potent regulator of endocan release. In increasing renal injury and macroalbuminuria, the decreased expression of VEGF-A, mainly secreted from podocytes and renal tubular cells, leads to decreased plasma endocan release. Furthermore, the authors stated that since endocan is an angiogenic molecule, it may be produced excessively in the early phase of diabetic nephropathy followed by a decline in its secretion in more advanced nephropathy [54]. Therefore, serum endocan levels may have a utility in the early detection of diabetic nephropathy, even before the development of micro- or macroalbuminuria. Thus, endocan may have a role as a marker in the monitoring of the progression of diabetic nephropathy.

### 3.5. Endocan Immunoglobulin a Nephropathy

Immunoglobulin A nephropathy (IgAN), the most common form of primary glomerulonephritis, characterized by the deposition of IgA-consisting immune complexes in the glomerulus, is a leading cause of CKD and renal failure [73]. Given that endothelial injury has been documented in several studies and that it is a key element in CKD progression, Lee et al. investigated the clinical relevance of plasma and urine endocan levels in patients with IgAN (*n* = 64) [51]. They observed that both plasma and urine endocan levels were significantly higher in patients with IgAN than in healthy controls. When the patients were grouped according to CKD stages, plasma endocan levels were not different among different CKD stages, but urine endocan levels were higher in patients with advanced CKD. When the patients were grouped according to the Lee grading system, both plasma and urine endocan levels were higher in patients with advanced pathologic grades. In contrast, there was no significant correlation between Oxford classification variables and plasma and urine endocan levels. Cox proportional hazard model analysis demonstrated that a high plasma endocan level was an independent risk factor for the rapid deterioration of renal function, which was unexpected as plasma endocan levels did not vary significantly among patients with different CKD stages. In contrast, urine endocan was not significantly associated with renal outcome. The authors concluded that the elevation of plasma endocan level might have a useful prognostic value of adverse renal outcome in IgAN patients because it could reflect vascular endothelial injury contributed to disease pathogenesis and CKD progression, unlike the increase of endocan in urine, which may be due to glomerular membrane damage. However, a limitation of the study pointed out was the intake of medications by some of the patients, such as lipid-lowering and antihypertensive drugs, which are known to influence endothelial activity and reduce plasma endocan levels [74, 75].

### 3.6. Endocan and Polycystic Kidney Disease

Autosomal dominant polycystic kidney disease (ADPKD) is the most common hereditary kidney disease [76]. It is characterized by the development of a multitude of renal cysts, which leads to massive enlargement of the kidney. Endothelial dysfunction has been previously identified as an important mechanism of renal injury progression in ADPKD [77]. Additionally, there is a significant angiogenic activity promoting a rich vascular network surrounding renal cysts in ADPKD. In this context, Raptis et al. sought to investigate the levels of emerging biomarkers of endothelial function, angiogenesis, and hypoxia, namely, endocan, angiopoietin-1, and hypoxia inducible factor-1a (HF-1a) in ADPKD patients [52]. The patient population consisted of a group of ADPKD individuals with modestly impaired renal function (*n* = 26) and a group of ADPKD individuals with relatively preserved renal function (*n* = 26). Patients with impaired renal function had significantly higher levels of endocan, angiopoietin-1 and HF-1a compared to both patients with preserved renal function and healthy controls. Modest inverse correlations of eGFR with the above markers were observed likewise in ADPKD patients. In order to explore a possible central role of endothelial dysfunction in determining levels of studied parameters, the authors examined correlations of asymmetric dimethylarginine (ADMA), an established marker of endothelial dysfunction with endocan, angiopoietin-1, and HF-1a in patients with ADPKD. The analysis showed a strong positive correlation between ADMA levels and endocan, as well as between ADMA and angiopoietin-1, and also ADMA and HF-1a. The authors suggested that endothelial dysfunction causing microvasculature changes linked to angiogenesis and hypoxia may come early in the course of ADPKD. Endocan combined with angiopoietin-1 and HF-1a could be emerging biomarkers for patients at the early stages, having potential utility for the assessment of the disease progression. Recently, Ekinci et al. conducted a study with 51 ADPKD and 20 hypertensive patients to evaluate endothelial dysfunction and atherosclerosis in terms of endocan, ADMA levels, flow-mediated dilatation (FMD), nitroglycerin-mediated dilation (NMD), and carotid intima-media thickness (CIMT) [78]. Although no significant difference was found in serum endocan levels between ADPKD and hypertensive patients, in ADPKD patients with eGFR≤60 ml/min/1.73 m^2^, serum endocan levels were significantly higher than those in patients with eGFR>60 ml/min/1.73 m^2^. Serum endocan levels were significantly elevated in ADPKD patients with nephrolithiasis compared to those without nephrolithiasis, while the other studied parameters were similar between the two groups. Besides, serum endocan levels were significantly higher in ADPKD patients with liver cysts compared to those without liver cysts, while no differences were detected for the other studied parameters between these subgroups.

AKI = acute kidney injury, ARDS = acute respiratory distress syndrome, pts = patients, cre = creatinine, ECR = endocan cleavage ratio: plasma p14/(endocan + p14), eGFR = estimated glomerular filtration rate, CKD = chronic kidney disease, FMV = flow-mediated vasodilation, CIMT = carotid intima thickness, CVE = cardiovascular events, CVD = cardiovascular disease, sICAM-1 = soluble intercellular adhesion molecule-1, sVCAM-1 = soluble vascular cell adhesion molecule-1, hs-CRP = high sensitivity C-reactive protein, HD = hemodialysis, TC = total cholesterol, LDL-C = low-density lipoprotein cholesterol, HDL-C = high-density lipoprotein cholesterol, PON = paraoxonase, HF = heart failure, KIM-1 = kidney injury molecule-1, NT-proBNP = N-terminal probrain natriuretic peptide, PD = peritoneal dialysis, PWV = pulse wave velocity, ESM-1 = endothelial cell specific molecule-1, ABMR = antibody-mediated rejection, HT = hypertension, DN = diabetic nephropathy, DM2 = diabetes mellitus type 2, IgAN = immunoglobulin A nephropathy, ADPKD = autosomal dominant polycystic kidney disease, ADMA = asymmetric dimethylarginine, NMD = nitroglycerin-mediated dilation.

## 4. Conclusions

Endocan is produced prevalently by endothelial cells of kidney vasculature. Its secretion by the activated endothelium and its regulatory effect in inflammation and endothelial dysfunction could explain the significant increase of plasma levels in several types of kidney diseases. Recent studies reveal that endocan could be a predictor of all-cause mortality and cardiovascular events in chronic kidney disease patients. It could be used additionally as a prognostic factor of the unfavorable cardiovascular outcome, especially in patients on peritoneal dialysis displaying rapid decline of urine output volume. Moreover, high plasma levels of endocan could be an independent prognostic factor for rapid renal function deterioration in IgA nephropathy. On the other hand, endocan levels could efficiently contribute to the diagnosis of several kidney disorders, such as acute kidney injury secondary to inflammation and endothelial damage. In cases of acute rejection and chronic renal allograft injury in renal transplant recipients, the detection of high serum levels of endocan could be used in the discrimination of antibody-mediated rejection from other etiologies of allograft dysfunction. However, the small size of subjects in the majority of the above-mentioned studies, the deprivation of direct data clarifying the origin of endocan plasma increase (increased production or decreased renal clearance), and the conflicting results pose limitations for the use of endocan concentration in clinical practice. Moreover, the cross-sectional design of most of the researches with the concomitant uncertainty about cause-and-effect associations might limit the strength of the findings. Larger scale, multicentered clinical trials focusing on the frequent follow-up and excluding the medications which interfere with endothelial integrity could provide a better insight into the applicability of endocan as a marker of prognosis and diagnosis in kidney diseases.

## Figures and Tables

**Table 1:** Benefits and limitations of endocan as a prognostic and diagnostic biomarker in kidney diseases.

**Benefits**
Soluble in human fluids
Having low concentration in physiological conditions
Easily measurable
Noninvasive
Multifunctional, associated with inflammation, endothelial injury, the decline of renal function, and/or incidence of cardiovascular events in several types of kidney disease

**Limitations**
Plasma elevation does not always correlate with kidney function deterioration
Several medications could influence its measurement
Large-scaled, multicentered clinical trials pend to define its applicability

**Table 2:** The most important findings concerning the role of endocan in kidney diseases.

Reference	Type of disease	Study groups	Key findings
Rahmania et al. [55]	AKI	96 pts with ARDS who were not receiving RRT at diagnosis	Plasma endocan levels in ARDS pts requiring RRT during hospitalization are higher than in those not needing RRT. Endocan and cre levels combined are most valuable in predicting the need for RRT
Gunay et al. [42]	AKI	39 pts with AKI	Endocan levels are higher in AKI pts than in NC. ROC analysis showed significant sensitivity and specificity in the identification of AKI
Gaudet et al. [56]	AKI	99 severe septic pts	Renal SOFA was the only component of the SOFA score associated with higher ECR. Circulating concentration of p14 might be influenced by the severity of AKI in septic pts
Yilmaz et al. [11]	CKD	251 pts with CKD (stage 1–5), not receiving dialysis	CKD pts displayed higher plasma endocan levels than controls, which correlated with lower eGFR and markers of inflammation and vascular abnormalities (FMV and CIMT). Plasma endocan levels were associated with all-cause of mortality and CVE independently of traditional risk factors
Bao et al. 2018 [57]	CKD	54 pts with CKD, stage 5, not receiving dialysis	Increased serum endocan is significantly associated with elevated night/day heart rate ratio in nondialysis stage 5 CKD pts
Perrotti et al. [58]	CKD	166 pts with preoperative CKD who underwent cardiac surgery	Measurement of plasma endocan in CKD pts after cardiac surgery predicts postoperative pulmonary infection with high sensitivity and specificity in a more time-saving way compared to clinical diagnosis
Pawlak et al. [10]	CKD	53 pts with CKD with and without CVD	Plasma endocan, soluble adhesion molecules sICAM-1/sVCAM-1, and hs-CRP were higher in pts with CKD and CVD than in CKD pts without CVD. There was no correlation between endocan levels and kidney function markers (eGFR, cre, and urea). In CKD pts with CVD, a positive correlation was observed between endocan and sICAM-1 and sVCAM
Samouilidou et al. [46]	CKD	105 pts with CKD, divided into nondialysis and HD subgroups	In CKD pts on HD, serum endocan levels were higher than in nondialyzed pts and controls. Endocan levels were significantly and positively correlated with TC and LDL-C in CKD, and additionally were negatively correlated with HDL-C in the HD group. Multiple regression analysis between endocan and lipid parameters including PON1 and PON3 fractions of PON, revealed that endocan was independently associated only with PON1 concentration
Mc Millan et al. [46]	CKD	90 pts with CKD-stage 5, on HD, with and without HF	In CKD pts with HD, there were no significant differences in plasma endocan levels of HD pts in stage 5 CKD with and without HF. In contrast to endocan, NT-proBNP and KIM-1 concentrations were significantly higher in HF (+)-CKD5-HD pts
Oka et al. [48]	CKD	21 CKD pts on PD	In PD patients, serum endocan levels were significantly increased in those belonging to the rapid decrease of the urine volume group, compared to the slow-volume group. Serum endocan was positively correlated with proteinuria level, serum cre, but not with urine volume at baseline
Poon et al. [47]	CKD	193 CKD pts on PD	PD patients having higher serum endocan levels had lower serum albumin, higher carotid-femoral PWV, and higher CRP. Serum endocan level was an independent predictor of cardiovascular-free survival
Li et al. [59]	CKD	60 pts who underwent RT, divided into a normally functioning renal allograft group and acute rejection subgroups	In patients with acute rejection, ESM-1 mRNA and protein expression peripheral blood increased significantly compared to patients with normal allograft function or dysfunction from other causes.
Lee et al. [12]	CKD	203 KT recipients with different etiologies of allograft dysfunction	In pts with acute ABMR, both plasma and urinary endocan levels were significantly higher than pts with normal pathology or other kidney etiologies. Pts with high urinary and plasma endocan levels in the ABMR group displayed significant worse renal survival and higher scores of microvascular inflammation in biopsy specimens
Su et al. [50]	CKD	97 RT recipients	Renal transplant pts with higher serum endocan levels displayed higher cre and lower eGFR levels than pts with lower endocan after 3 months of follow-up
Malyszko et al. [49]	CKD	63 RT recipients	Endocan level correlated positively with other markers of endothelial damage (ICAM-1, VCAM-1) and negatively with eGFR and time after transplantation
De Souza et al. [60]	CKD	62 pediatric renal transplants with and without HT	In pediatric pts with HT and loss of renal function, serum endocan levels were significantly elevated and inversely correlated with eGFR. An endocan cutoff concentration of 70 ng/mL identified pts who had HT and loss of renal function with 100% sensitivity and 75% specificity
Ekiz-Bellir et al. [53]	DN	96 DM2 pts, classified according to their 24 h urinary albumin excretion rate	In DM2 pts, endocan and endoglin serum levels were significantly higher than in controls. Endocan levels of DN pts were higher than those of normoalbuminuric pts, but there was no significant difference in endoglin levels between these groups
Cikrikcioglu et al. [54]	DN	137 DM2 pts, divided into normoalbuminuria, microalbuminuria and macroalbuminuria groups	Pts with DM2 and macroalbuminuria had significantly lower serum endocan levels than normoalbuminuric and microalbuminuric pts. Urine albumin-cre ratio (UACR) and urine protein-cre ratio (UPCR) displayed negative correlation with endocan levels in pts with macroalbuminuria
Lee et al. [51]	IgAN	64 pts with IgAN	In pts with IgAN, both plasma and urine endocan levels were significantly higher than in controls. Plasma endocan levels were not significantly different across CKD stages; however, the urine endocan levels increased in advanced CKD stages. Cox proportional hazard models showed that high plasma endocan was an independent risk factor for CKD progression
Raptis et al. [52]	ADPKD	26 ADPKD pts with preserved renal function in comparison to 26 ADPKD pts with impaired renal function	Pts with ADPKD with impaired renal function had significantly increased levels of endocan in comparison to ADPKD with preserved renal function and controls. A strong correlation was observed between ADMA and endocan, as well as between ADMA and angiopoietin-2
Ekinci et al. [78]	ADPKD	51 ADPKD pts	In ADPKD pts, serum endocan, CIMT, and ADMA levels were higher, whereas NMD was lower in pts with eGFR≤60 mL/min/1.73 m^2^ than pts with eGFR>60 mL/min/1.73 m^2^

## Data Availability

The data used to support this article will be provided upon request from the corresponding author.

## References

[1] Lassalle P., Molet S., Janin A. (1996). ESM-1 is a novel human endothelial cell-specific molecule expressed in lung and regulated by cytokines. *Journal of Biological Chemistry*.

[2] Béchard D., Gentina T., Delehedde M. (2001). Endocan is a novel chondroitin sulfate/dermatan sulfate proteoglycan that promotes hepatocyte growth factor/scatter factor mitogenic activity. *Journal of Biological Chemistry*.

[3] Bechard D., Meignin V., Scherpereel A. (2000). Characterization of the secreted form of endothelial-cell-specific molecule 1 by specific monoclonal antibodies. *Journal of Vascular Research*.

[4] Mikkelsen M. E., Shah C. V., Scherpereel A. (2012). Lower serum endocan levels are associated with the development of acute injury after major trauma. *Journal of Critical Care*.

[5] Tang l, Zhao Y., Wang D. (2014). Endocan levels in peripheral blood predict outcomes of acute respiratory distress syndrome. *Mediators of Inflammation*.

[6] Güzel A., Duran L., Köksal N. (2014). Evaluation of serum endothelial cell specific molecule-1 (endocan) levels as a biomarker in patients with pulmonary thromboembolism. *Blood Coagulation & Fibrinolysis: An International Journal in Haemostasis and Thrombosis*.

[7] Xiong C., Zhao Z.-w., Chen Z.-y. (2015). Elevated human endothelial cell-specific molecule-1 level and its association with coronary artery disease in patients with hypertension. *Journal of Investigative Medicine*.

[8] Balta S., Mikhailidis D. P., Demirkol S., Ozturk C., Celik T., Iyisoy A. (2015). Endocan: a novel inflammatory indicator in cardiovascular disease?. *Atherosclerosis*.

[9] Emet S., Elitok A., Onur I. (2017). Endocan: a novel biomarker associated with well-developed coronary collateral circulation in patients with stable angina and chronic total occlusion. *Journal of Thrombosis and Thrombolysis*.

[10] Pawlak K., Mysliwiec M., Pawlak D. (2015). Endocan - the new endothelial activation marker independently associated with soluble endothelial adhesion molecules in uraemic patients with cardiovascular disease. *Clinical Biochemistry*.

[11] Yilmaz M. I., Siriopol D., Saglam M. (2014). Plasma endocan levels associate with inflammation, vascular abnormalities, cardiovascular events, and survival in chronic kidney disease. *Kidney International*.

[12] Lee Y. H., Kim S.-Y., Moon H. (2019). Endocan as a marker of microvascular inflammation in kidney transplant recipients. *Scientific Reports*.

[13] Scherpereel A., Depontieu F., Grigoriu B. (2005). Endocan, a new endothelial marker in human sepsis. *Critical Care Medicine*.

[14] Gaudet A., Parmentier E., Dubucquoi S. (2018). Low endocan levels are predictive of acute respiratory distress syndrome in severe sepsis and septic shock. *Journal of Critical Care*.

[15] Scherpereel A., Gentina T., Grigoriu B. (2003). Overexpression of endocan induces tumor formation?. *Cancer Research*.

[16] Delehedde M., Devenyns L., Maurage C. A., Vivès R. R. (2013). Endocan in cancers: a lesson from a circulating dermatan sulfate proteoglycan. *International Journal of Cell Biology*.

[17] Huang X., Chen C., Wang X. (2016). Prognostic value of endocan expression in cancers: evidence from meta-analysis. *OncoTargets and Therapy*.

[18] Sarrazin S., Lyon M., Deakin J. A. (2010). Characterization and binding activity of the chondroitin/dermatan sulfate chain from endocan, a soluble endothelial proteoglycan. *Glycobiology*.

[19] Kechagia M., Papassotiriou I., Gourgoulianis K. I. (2016). Endocan and the respiratory system: a review. *International Journal of Chronic Obstructive Pulmonary Disease*.

[20] Zhang S., Zuo L., Zhou Q. (2012). Expression and distribution of endocan in human tissues. *Biotechnic & Histochemistry*.

[21] Bessa J., Albino-Teixeira A., Reina-Couto M., Sousa T. (2020). Endocan: a novel biomarker for risk stratification, prognosis and therapeutic monitoring in human cardiovascular and renal diseases. *Clinica Chimica Acta*.

[22] Kali A., Shetty K. S. R. (2014). Endocan: a novel circulating proteoglycan. *Indian Journal of Pharmacology*.

[23] De Freitas Caires N., Gaudet A., Portier L., Tsicopoulos A., Mathieu D., Lassalle P. (2018). Endocan, sepsis, pneumonia, and acute respiratory distress syndrome. *Critical Care*.

[24] Maurage C.-A., Adam E., SarrazinMinéo S. (2009). Endocan expression and localization in human glioblastomas. *Journal of Neuropathology and Experimental Neurology*.

[25] Rennel E., Mellberg S., Dimberg A. (2007). Endocan is a VEGF-A and PI3K regulated gene with increased expression in human renal cancer. *Experimental Cell Research*.

[26] Sun H., Zhang H., Li K. (2018). ESM‐1 promotes adhesion between monocytes and endothelial cells under intermittent hypoxia. *Journal of Cellular Physiology*.

[27] Lee W., Ku S.-K., Kim S.-W., Bae J.-S. (2014). Endocan elicits severe vascular inflammatory responses in vitro and in vivo. *Journal of Cellular Physiology*.

[28] Tadzic R., Mihalj M., Vcev A., Ennen J., Tadzic A., Drenjancevic I. (2013). The effects of arterial blood pressure on endocan and soluble endothelial cell adhesion molecules (CAMs) and CAMs ligands expression in hypertensive patients on Ca blocker therapy. Kidney. *Kidney and Blood Pressure Research*.

[29] Zhang H., Shen Y.-W., Zhang L.-J. (2021). Targeting endothelial cell-specific molecule 1 protein in cancer: a promising therapeutic approach. *Frontiers in Oncology*.

[30] Grigoriu B. D., Depontieu F., Scherpereel A. (2006). Endocan expression and relationship with survival in human non-small cell lung cancer. *Clinical Cancer Research*.

[31] Atukeren P., Kunbaz A., Turk O. (2016). Expression of endocan in patients with meningiomas and gliomas. *Disease Markers*.

[32] Leroy X., Aubert S., Zini L. (2010). Vascular endocan (ESM-1) is markedly overexpressed in clear renal cell carcinoma. *Histopathology*.

[33] Chen L.-Y., Liu X., Wang S.-L., Qin C.-Y. (2010). Overexpression of the endocan gene in endothelial cells from hepatocellular carcinoma is associated with angiogenesis and tumor invasion. *Journal of International Medical Research*.

[34] Huang G.-W., Tao Y.-M., Ding X. (2009). Endocan expression correlated with poor survival in human hepatocellular carcinoma. *Digestive Diseases and Sciences*.

[35] Matano F., Yoshida D., Ishii Y., Tahara S., Teramoto A., Morita A. (2014). Endocan, a new invasion and angiogenesis marker of pituitary adenomas. *Journal of Neuro-Oncology*.

[36] Roudnicky F., Poyet C., Wild P. (2013). Endocan is upregulated in invasive bladder cancer where it mediates VEGF-A-induced angiogenesis. *Cancer Research*.

[37] Dieterich L. C., Mellberg S., Langenkamp E. (2012). Transcriptional profiling of human glioblastoma vessels indicates a key role of VEGF-A and TGF beta 2 in vascular abnormalization. *The Journal of Pathology*.

[38] Del Toro R., Prahst C., Mahivet T. (2010). Identification and functional analysis of endothelial tip cell-enriched genes. *Blood*.

[39] Malyszko J. (2010). Mechanism of endothelial dysfunction in chronic kidney disease. *Clinica Chimica Acta*.

[40] Verma S. K., Molitoris B. A. (2015). Renal endothelial injury and microvascular dysfunction in acute kidney injury. *Seminars in Nephrology*.

[41] Wu-Wong J. R. (2008). Endothelial dysfunction and chronic kidney disease: treatment options. *Current Opinion in Investigational Drugs*.

[42] Gunay M., Mertoglu C. (2019). Increase of endocan, a new marker of inflammation and endothelial dysfunction, in acute kidney injury. *North Clin Instanb*.

[43] Azimi A. (2017). Could “calprotectin” and “endocan” serve as “Troponin of Nephrologists”?. *Medical Hypotheses*.

[44] Afsar B., Takir M., Kostek O., Covic A., Kanbay M. (2014). Endocan: a new molecule playing a role in the development of hypertension and chronic kidney disease. *Journal of Clinical Hypertension*.

[45] Lee H. G., Choi H. Y., Bae J.-S. (2014). Endocan as a potential diagnostic or prognostic biomarker for chronic kidney disease. *Kidney International*.

[46] Samouilidou E., Bountou E., Papandroulaki F., Papamanolis M., Papakostas D., Grapsa E. (2018). Serum endocan levels are associated with paraoxonase 1 concentration in patients with chronic kidney disease. *Therapeutic Apheresis and Dialysis: Official Peer-Reviewed Journal of the International Society for Apheresis, the Japanese Society for Apheresis, the Japanese Society for Dialysis Therapy*.

[47] Poon P. Y. K., Chung Ng J. K., Wing-Shun Fung W. (2018). Relationship between plasma endocan level and clinical outcome of Chinese peritoneal dialysis patients. *Kidney Blood Pressure*.

[48] Oka S., Obata Y., Sato S. (2017). Serum endocan as a predictive marker for decreased urine volume in peritoneal dialysis patients. *Medical Science Monitor*.

[49] Malyszco J., Zorawska E., Malyszco J. (2018). Endocan concentration in kidney transplant recipients. *Transplantation Proceedings*.

[50] Su Y.-H., Shu K.-H., Hu C.-P. (2014). Serum endocan correlated with stage of chronic kidney disease and deterioration in renal transplant recipients. *Transplantation Proceedings*.

[51] Lee Y. H., Kim J. S., Kim S.-Y. (2016). Plasma endocan level and prognosis of immunoglobulin A nephropathy. *Kidney Research and Clinical Practice*.

[52] Raptis V., Bakogiannis C., Loutradis C. (2018). Levels of endocan, angiopoietin-2, and hypoxia-inducible factor-1a in patients with autosomal dominant polycystic kidney disease and different levels of renal function. *American Journal of Nephrology*.

[53] Ekiz-Billir B., Billir B., Aydin M., Soysal-Atile N. (2019). Evaluation of endocan and endoglin levels in chronic kidney disease due to diabetes mellitus. *Archives of Medical Science*.

[54] Cikrikcioglu M. A., Erturk Z., Kilic E. (2016). Endocan and albuminuria in type 2 diabetes mellitus. *Renal Failure*.

[55] Rahmania L., Orbegozo Cortés D., Irazabal M. (2015). Elevated endocan levels are associated with the development of renal failure in ARDS patients. Intensive. *Care Med Exp*.

[56] Gaudet A., Parmentier E., De Freitas Caires N. (2019). Impact of acute renal failure on plasmatic levels of cleaved endocan. *Critical Care*.

[57] Bao Y., Wang Y. A., Xiao H. (2018). Serum endocan and circadian heart rate variability in nondialysis stage 5 chronic disease patients. *International Urology and Nephrology*.

[58] Perrotti A., Chenevier-Gobeaux C., Ecarnot F. (2017). Relevance of endothelial cell-specific molecule 1 (endocan) plasma levels for predicting pulmonary infection after cardiac surgery in chronic kidney disease patients: the Endolung pilot study. *Cardiorenal Medicine*.

[59] Li S., Wang L., Wang C. (2012). Detection of endothelial cell specific molecule-1 in acute rejection after renal transplantation. *Urology*.

[60] De Souza L. V., Oliviera V., Laurindo A. O., Huarachi D. R. G., Nogueira P. C. K., de Santis Feltran L. (2016). Serum endocan levels associated with hypertension and loss of renal function in pediatric patients after two years from renal transplant. *The Internet Journal of Nephrology*.

[61] KDIGO AKI Work Group (2012). KDIGO clinical practice guideline for acute kidney injury. *Kidney International-Supplement*.

[62] KDIGO Diabetes Work Group (2020). KDIGO 2020 Clinical practice guideline for diabetes management in chronic kidney disease. *Kidney International-Supplement*.

[63] Diaz-Ricard M., Torramade-Moix S., Pascual G. (2020). Endothelial damage, inflammation and immunity in chronic kidney disease. *Toxins*.

[64] Nalewajska M., Gurazda K., Marchelek-Myśliwiec M., Pawlik A., Dziedziejko V. (2020). The role of endocan in selected kidney diseases. *International Journal of Molecular Sciences*.

[65] Beddhu S., Nigwekar S. U., Ma X., Greene T. (2009). Associations of resting heart rate with insulin resistance, cardiovascular events and mortality in chronic kidney disease. *Nephrology Dialysis Transplantation*.

[66] McMillan R., Skiadopoulos L., Hoppensteadt D. (2018). Biomarkers of endothelial, renal, and platelet dysfunction in stage 5 chronic kidney disease hemodialysis patients with heart failure. *Clinical and Applied Thrombosis*.

[67] Herget-Rosenthal S., von Ostrowski M., Kribben A. (2012). Definition and risk factors of rapidly declining residual renal function in peritoneal dialysis. An observational study. *Kidney and Blood Pressure Research*.

[68] Dahle D. O., Midtvedt K., Hartmann A. (2013). Endothelial dysfunction is associated with graft los in renal transplant recipients. *Transplantation*.

[69] Mitsnefes M. M., Khoury P. R., McEnery P. T. (2003). Early post transplantation hypertension and poor long-term renal allograft survival in pediatric patients. *The Journal of Pediatrics*.

[70] Arman Y., Akpinar T. S., Kose M. (2016). Effect of glycemic regulation on endocan levels in patients with diabetes: a preliminary study. *Angiology*.

[71] Tian H., Huang J. J., Golzio C. (2018). Endoglin interacts with VEGFR2 to promote angiogenesis. *The FASEB Journal*.

[72] Wakelin S. J., Marson L., Howie S. E., Garden J., Lamb J. R., Forsythe J. L. (2004). The role of vascular endothelial growth factor in the kidney in health and disease. *Nephron Physiology*.

[73] Rodrigues J. C., Haas M., Reich H. N. (2017). IgA nephropathy (review). *Clinical Journal of the American Society of Nephrology*.

[74] Tziomalos K., Athyros V., Karagiannis A., Mikhailidis D. (2012). Lipid-lowering agents and the endothelium: an update after 4 years. *Current Vascular Pharmacology*.

[75] Celik T., Balta S., Karaman M. (2015). Endocan, a novel marker of endothelial dysfunction in patients with essential hypertension: comparative effects of amlodipine and valsartan. *Blood Pressure*.

[76] Comec-Le Gall E., Alam A., Perrone R. (2019). Autosomal dominant polycystic disease. *Lancet*.

[77] Raptis V., Loutradis C., Sarafidis P. A. (2018). Renal injury progression in autosomal dominant polycystic kidney disease: a look beyond the cysts. *Nephrology Dialysis Transplantation*.

[78] Ekinci I., Buyukkaba M., Cinar A. (2021). Endothelial dysfunction and atherosclerosis in patients with autosomal dominant polycystic kidney disease. *Cue*.

